# Prevalence of Sarcopenia and Its Relationship with Sites of Fragility Fractures in Elderly Chinese Men and Women

**DOI:** 10.1371/journal.pone.0138102

**Published:** 2015-09-14

**Authors:** Wei Hong, Qun Cheng, Xiaoying Zhu, Hanmin Zhu, Huilin Li, Xuemei Zhang, Songbai Zheng, Yanping Du, Wenjing Tang, Sihong Xue, Zhibin Ye

**Affiliations:** 1 Department of Osteoporosis, Research Section of Geriatric Metabolic Bone Disease, Huadong Hospital affiliated to Fudan University, Shanghai, China; 2 Department of Nephrology, Huadong Hospital affiliated to Fudan University, Shanghai, China; Nanjing Medical University, CHINA

## Abstract

**Objective:**

Sarcopenia might be associated with bone fragility in elderly individuals. This study aimed to investigate the prevalence of sarcopenia and its association with fragility fracture sites in elderly Chinese patients.

**Methods:**

Patients (322 men and 435 women) aged 65–94 years and with a history of fragility fractures in the ankle, wrist, vertebrae or hip, and healthy men (n = 1263) and women (n = 1057) aged 65–92 years without a history of fractures were enrolled. Whole-body dual energy X-ray absorptiometry was used to analyze skeletal muscle mass index (SMI), fat mass and bone mineral density. Sarcopenia was defined as SMI less than two standard deviations below the mean of a young reference group.

**Results:**

Sarcopenia occurrence varied with fracture location. Sarcopenia was more common in females with vertebral and hip fractures and in men with hip and ankle fractures than in the non-fracture group). Sarcopenia was significantly more prevalent in men with wrist, hip and ankle fractures than in women. SMI was correlated with BMD in different fracture groups. Logistic regression analyses revealed that lower SMI was associated with an increased risk of hip fracture both in men and women and ankle fracture in men.

**Discussion:**

Sarcopenia may be an independent risk factor for hip and ankle fractures in men, and for hip fractures in women.

## Introduction

A typical feature of aging within the musculoskeletal system is “loss”: loss of bone mass, muscle mass and strength, and hormone production. All these changes increase the risk of fragility fractures.[1] As the general population is aging in China, fragility fracture incidence is increasing rapidly.[2] Loss of skeletal muscle mass and strength accelerates after the age of 65.[3] Muscle loss is associated with mobility disorders, increased risk of falls, reduced ability to function in daily living activities, loss of independence, and reduced life expectancy.[4] Sarcopenia is an age-related decline in muscle bulk and quality that is associated with the development of frailty.[5] Furthermore, the loss of muscle tissue seems strictly associated with the loss of bone mass and strength.[3, 6] Bone mass and muscle mass are both regulated by a variety of common hormones and genetic factors, which have simultaneous physical and mechanical effects.[7] Several clinical studies have noted that sarcopenia coexists with both osteoporosis and osteopenia.[8] The loss of muscle mass is accompanied by loss of bone and sarcopenia in osteoporosis patients, and is significantly more common than in subjects with normal bone mineral density (BMD).[6, 9]

To date, sarcopenia diagnosis is carried out according to two distinct standards. Baumgartner’s criteria defined sarcopenia as a value of appendicular skeletal muscle mass two standard deviations below the average value calculated in healthy, young men and women.[4] On the other hand, the European Society for Clinical Nutrition and Metabolism (ESPEN) defined sarcopenia as a condition characterized by loss of muscle mass and strength.[3, 10] Appendicular skeletal muscle mass can be measured accurately using computed tomography (CT) or magnetic resonance imaging (MRI). However, the costs, availability and, for CT, radiation exposure restrict their use. In clinical practice, dual-energy X-ray absorptiometry (DXA) is precise and more accessible for measuring muscle mass.[11] Indeed, the DXA approach yielded total body skeletal muscle estimates similar to values measured by CT, although DXA tended to overestimate total body skeletal muscle by 5.1–5.8%.[12]

Fragility fractures have become a major public health concern because they result in increased mortality, persistent physical morbidity and limit every-day activities.[13] Osteoporosis and falls are the most important risks factors of fragility fracture.[14] The most common location of fragility fractures are the wrist, ankle, vertebrae and hip.[15] Sarcopenia is considered an important risk factor for falling.[4, 12] A high prevalence of sarcopenia and reduced leg muscle mass was seen in Japanese patients immediately after a hip fracture.[16] We previously evaluated the loss of muscle mass corresponding to sarcopenia among Chinese men and women and established sarcopenia reference values.[17] However, the impact of sarcopenia on other fracture locations has been rarely reported. We hypothesized that sarcopenia might be associated with bone fragility, especially in elderly individuals. The aim of the present study was to investigate the prevalence of sarcopenia and the association between sarcopenia and different types of fragility fractures in elderly Chinese patients.

## Methods

### Subjects

Patients with a history of fragility fractures were selected for inclusion at the Shanghai Huadong Hospital, affiliated with Fudan University between January 2006 and December 2012. During the same period, healthy controls without a history of fragility fracture that fulfilled the other inclusion and exclusion criteria were also selected from individuals undergoing physical examinations at the Shanghai Huadong Hospital.

The inclusion criteria were: 1) patients with a history of fragility fracture in the ankle, wrist, vertebrae or unilateral hip; 2) aged over 65 years; and 3) in good health according to medical evaluation. A fragility fracture was defined according to the World Health Organization (WHO) as a fracture caused by an injury that would be insufficient to fracture a normal bone, such as a fall from a standing height or less, or from no identifiable trauma.[18]

The exclusion criteria were: 1) individuals who were confined to a wheelchair or a bed; 2) individuals with chronic diseases, such as hyperthyroidism, hyperparathyroidism, renal failure, malabsorption syndrome, alcoholism, chronic colitis, multiple myeloma, leukemia, chronic arthritis, stroke, Parkinson’s disease, peripheral neuropathy, or cognitive impairment; 3) medications that were likely to affect bone or soft tissue metabolism, such as antiosteoporotic (e.g. glucocorticoids, heparin, warfarin, thyroxine, sex hormones, bisphosphonate, selective estrogen modulators, calcitonin, parathyroid hormone analogue, or calcitriol) or weight-controlling drugs; or 4) diets for weight loss or weight gain.

The fragility fracture group included 757 patients (322 men and 435 women, aged 65–94 years). The healthy control non-fracture group included 2320 individuals (1263 men and 1057 women, aged 65–92). All subjects provided a written informed consent before participating in the study. The program was approved by the ethics committee of Huadong Hospital affiliated to Fudan University.

### Measurements and methods

Body weight was measured without shoes. Body mass index (BMI) was calculated as weight (kg) divided by height squared (m^2^). Regional BMD, including lumbar spine (L1-4) and contralateral non-fracture femoral neck (FN) were measured using DXA (Hologic Delphi A; Hologic, Inc., Bedford, MA, USA) using the APEX System software v3.1. Body composition was measured with a whole body scan using the same DXA machine operated in slow scan mode. DXA scans were analyzed with manual DXA analysis software. All scans and analyses were conducted by the same investigator. Day-to-day coefficients of variance (CVs) of these observations were 0.86% in the lumbar spine BMD, 1.86% in the FN BMD, 0.95% in the total body BMD, 0.74% in lean mass, and 1.5% in fat mass. The densitometer was standardized by a standard phantom prior to each measurement. Sarcopenia was based on appendicular lean mass (ALM/kg^2^) measurements.[19] The cutoff values to define class 1 and 2 sarcopenia in each gender were respectively defined as one and two standard deviations (SD) below the sex-specific means of the reference data for young people aged 18–40 years in our previous study.[17] Class 1 and 2 sarcopenia were skeletal muscle mass index (SMI) below 7.01 and 6.08 kg/m^2^, respectively, in Chinese men and 5.42 and 4.79 kg/m^2^, respectively, in Chinese women. According to the WHO definition [20] and the BMD reference data established by young people aged in their twenties in this study, subjects with a BMD that was 2.5 SD lower than the peak mean of the same gender (T score B-2.5) were determined to be osteoporotic.

Obesity was defined percentage body fat greater than 25% in men and 35% in women. osteosarcopenic obesity was defined at study baseline as appendicular skeletal muscle mass divided by stature squared <7.01 kg/m^2^ in men and 6.08 kg/m^2^ in women with obesity having L1–L4 and/or total femur T scores below−1 [21, 22].

### Statistical analysis

Analyses were performed using SPSS 17.0 for Windows (IBM, Armonk, NY, USA). Continuous variables are expressed as means ± SDs. Differences in basic characteristic were evaluated using analysis of variance (ANOVA). The chi-squared test was used for the comparison of the prevalence of sarcopenia between men and women, and was also used in the different types of fracture groups. Pearson’s correlation was used to investigate the relationship with BMD and sarcopenia. To determine the risk factors for predicting fragility fracture expect of BMD, we used a binary logistic regression model adjusted for LSBMD and FNBMD. All statistical tests were two-tailed, and P<0.05 was considered significant.

## Results

Table 1 shows the patients’ background characteristics, body composition and skeletal muscle mass index. All patients were aged from 65 to 94 years old. In women, SMI was lower in the vertebral fracture (VF) and hip fracture (HF) groups compared with the non-fracture (NF) group. LSBMD and FNBMD were lower in all groups compared with the NF group, except the FNBMD in the waist fracture (WF) group. However, there was a statistical difference in total BMD only in the VF group. LM and FM did not differ much in most groups (only TLM was lower in the VF and HF groups). However, interestingly, the TFM and Fat% were higher in the AF group.

**Table 1:** Characteristics of the patients according to fracture groups and gender.

Characteristics	Wrist fracture		Vertebral fracture		Hip fracture		Ankle fracture		Non-fracture	
	Female	Male	Female	Male	Female	Male	Female	Male	Female	Male
n	106	67	112	110	93	69	124	76	1057	1263
Age (years)	73.57±6.14	76.00±6.71	77.98±7.54	80.72±6.63	79.87±6.86	82.28±5.76	74.93±6.89	78.92±7.08	77.26±6.52 a d	78.71±5.98^a^ ^b^ ^c^
Height (m)	1.53±0.06	1.66±0.64	1.51±0.07	1.65±0.06	1.54±0.06	1.63±0.05	1.54±0.05	1.65±0.06	1.53±0.05	1.67±0.49^a^ ^b^ ^c^ ^d^
Weight (kg)	57.46±9.29	70.2±10.18	55.49±8.80	67.80±10.39	54.08±7.84	57.34±8.40	61.47±7.43	69.39±11.90	59.11±9.20^b^ ^c^	72.75±6.36^a^ ^b^ ^c^ ^d^
BMI (kg/m^2^)	24.43±3.26	25.12±2.82	24.29±3.47	24.75±2.89	23.78±2.95	21.47±3.10	25.89±3.30	24.99±3.08	25.33±3.60^c^ ^d^	25.89±2.42^b^ ^c^ ^d^
LSBMD (g/cm^2^)	0.80±0.13	0.94±1.59	0.79±0.16	0.91±0.23	0.77±0.15	0.85±0.13	0.82±0.14	0.99±0.21	0.89±0.17^a^ ^b^ ^c^ ^d^	1.06±0.19^a^ ^b^ ^c^ ^d^
FNBMD (g/cm^2^)	0.60±0.09	0.69±0.13	0.56±0.09	0.63±0.11	0.52±0.14	0.57±0.10	0.57±0.09	0.69±0.11	0.60±0.09^b^ ^c^ ^d^	0.72±0.11^a^ ^b^ ^c^ ^d^
Total hip BMD (g/cm^2^)	0.73±0.11	0.86±0.16	0.67±0.13	0.78±0.14	0.58±0.08	0.69±0.13	0.69±0.12	0.85±0.15	0.72±0.12^b^ ^c^ ^d^	0.90±0.12^a^ ^b^ ^c^ ^d^
TBMD (g/cm^2^)	0.93±0.10	1.05±0.13	0.90±0.11	0.99±0.10	0.91±0.14	0.98±0.15	0.91±0.09	1.03±0.11	0.93±0.09^b^	1.08±0.10^b^ ^c^ ^d^
Arm LM (kg)	3.42±0.60	5.22±0.90	3.27±0.55	4.99±0.69	3.30±0.45	4.27±0.54	3.50±0.44	4.83±0.73	3.44±0.53^b^	5.18±0.63^b^ ^c^ ^d^
Trunk LM (kg)	18.58±3.35	25.59±3.57	17.91±2.23	24.95±3.27	18.74±2.16	21.58±2.84	19.22±2.13	24.53±3.38	19.25±2.49^b^	26.30±2.82^a^ ^b^ ^c^ ^d^
Leg LM (kg)	10.44±3.19	15.34±2.47	10.26±1.65	14.76±2.22	8.73±3.73	12.26±1.63	10.93±2.35	14.38±2.30	11.04±1.59^c^	15.48±1.83^b^ ^c^ ^d^
TLM (kg)	36.85±5.06	50.10±6.86	34.75±4.39	48.68±6.25	35.66±3.85	41.74±4.72	37.03±4.07	47.52±6.49	37.11±4.52^b^ ^c^	50.99±4.95^b^ ^c^ ^d^
Arm FM (kg)	2.45±0.72	1.90±0.57	2.47±0.84	1.83±0.53	2.42±0.66	1.45±0.58	2.75±0.72	1.93±0.59	2.56±0.74^d^	2.16±0.54^a^ ^b^ ^c^ ^d^
Trunk FM (kg)	10.24±3.93	10.11±3.22	9.80±3.46	9.71±3.33	9.90±3.01	6.90±2.99	12.02±3.05	10.43±3.48	10.88±3.32^d^	11.50±2.91^a^ ^b^ ^c^ ^d^
Leg FM (kg)	6.37±1.65	4.98±1.38	6.20±1.82	4.83±1.13	6.09±2.02	4.28±1.43	6.86±1.89	5.05±1.58	6.31±2.12^c^	5.57±1.54^a^ ^b^ ^c^ ^d^
TFM (kg)	19.43±5.39	18.15±4.94	19.46±5.87	17.44±5.03	18.74±5.16	13.72±4.79	22.76±5.04	18.54±5.31	20.93±5.54^d^	20.43±4.46^a^ ^b^ ^c^ ^d^
Fat%	33.32±5.16^d^	25.36±4.80	33.95±6.55^d^	25.52±4.98	33.89±4.76^d^	23.72±5.61	36.45±4.33	26.62±4.17	34.55±5.19^d^	27.31±6.67^a^ ^b^ ^c^
ALM (kg)	14.00±2.36	20.59±3.31	13.54±2.15	19.76±2.81	13.59±1.89	16.52±2.05	14.53±1.82	19.11±2.94	14.68±2.04^b^ ^c^	20.66±2.34^b^ ^c^ ^d^
SMI (kg/m^2^)	6.01±0.79	7.26±0.91	5.89±0.78	7.11±0.75	5.68±0.73	6.19±0.77	6.17±0.73	6.96±0.85	6.27±0.74^b^ ^c^	7.28±0.97^c^ ^d^

Abbreviations: BMI = body mass index; LSBMD = lumbar spine bone mineral density; FNBMD = femoral neck bone mineral density; BMD = bone mineral density; TBMD = total body bone mineral density; LM = lean mass; TLM = total lean mass; FM = fat mass; TFM = trunk fat mass; Fat% = (TFM/weight)*100; ALM = appendicular lean mass; SMI = skeletal muscle index

^a^: P<0.05 compared with wrist fracture group.

^b^: P<0.05 compared with vertebral fracture group

^c^: P<0.05 compared with hip fracture group.

^d^: P<0.05 compared with ankle fracture group

In men, SMI was lower in the HF and ankle fracture (AF) groups, and LM and FM were also lower. Almost all variables in all groups were significantly lower, except total BMD and LM in the WF group.

Table 2 shows the prevalence of sarcopenia in the four types of fracture for men and women. According to the cutoff values [17], the prevalence of class 1 and 2 sarcopenia was 20.8% and 3.8% in the female WF group, and 12.9% and 8.1% in the female AF group, compared with the prevalence of class 1 and 2 sarcopenia in the female NF group (14.4% and 3.4%) (all P>0.05). The prevalence of class 1 and 2 sarcopenia were 21.4% and 12.5% in the female VF group and 25.8% and 16.1% in the female HF group, respectively (both P<0.001 vs. the NF group).

**Table 2:** Prevalence of sarcopenia according to fracture groups and gender.

	Wrist fracture		Vertebral fracture		Hip fracture		Ankle fracture		Non-fracture	
	Female	Male	Female	Male	Female	Male	Female	Male	Female	Male
n	n = 106	n = 67	n = 112	n = 110	n = 93	n = 69	n = 124	n = 76	n = 1057	n = 1263
Sarcopenia									b c	c d
Normal	80 (75.5%)	38 (56.7%)	74 (66.1%)	66 (60.0%)	54 (58.1%)	10 (14.5%)	98 (79.0%)	38 (50.0%)	869 (82.2%)	783 (62.0%)
Class 1	22 (20.8%)	21 (31.3%)	24 (21.4%)	36 (32.7%)	24 (25.8%)	21 (30.6%)	16 (12.9%)	25 (32.9%)	152 (14.4%)	404 (32.0%)
Class 2	4 (3.8%)	8 (11.9%)	14 (12.5%)	8 (7.3%)	15 (16.1%)	37 (54.9%)	10 (8.1%)	13 (17.1%)	36 (3.4%)	76 (6.0%)
Total sarcopenia (class 1 + class 2)	26 (24.5%)	29 (43.3%)	38 (33.9%)	44 (40.0%)	39 (41.9%)	58 (84.1%)	26 (21.0%)	38 (50%)	188 (17.8%)	480 (38.0%)
Obesity										
Obesity	31(29.8%)	28(41.8%)	32(30.5%)	44(40.0%)	27(28.0%)	23(33.3%)	51(41.1%)	35(46.1%)	342(32.4%)^d^	554(43.8%)
Osteosarcopenic obesity (OSO)	6(5.7%)	7(10.4%)	9(8.0%)	14(12.7%)	7(7.5%)	16(23.2%)	12(9.6%)	12(15.8%)	46(4.4%)^d^	84(6.7%)^b^ ^c^ ^d^

^a^: P<0.05compared with wrist fracture group

^b^: P<0.05 compared with vertebral fracture group

^c^: P<0.05compared with hip fracture group

^d^: P<0.05compared with ankle fracture group

The prevalence of class 1 and 2 sarcopenia was 31.3% and 11.9% in the male WF group, and 32.7% and 7.3% in the male VF group (all P>0.05). The prevalence of class 1 and 2 sarcopenia were 30.6% and 54.9% in the male HF group and 32.9% and 17.1% in the male AF group (both P<0.001 vs. the male NF group). The prevalence of sarcopenia was significantly higher in men in the WF, HF, and AF groups compared with women (P<0.001). The prevalence of obesity in AF group in women was significantly higher in women than NF group. The prevalence of osteosarcopenic obesity in VF, HF, AF group in men and AF in women were higher than the NF group (all P<0.05).

In order to investigate the relationship between BMD and sarcopenia in different fracture groups, the Pearson’s correlation analysis was performed (Table 3). LSBMD was correlated with SMI only in the men VF group (r = 0.439, P<0.01). FNBMD was correlated with SMI in the women WF (r = 0.270, P<0.01), VF (r = 0.159, P<0.05) and HF (r = 0.337, P<0.01) groups, and in men VF (r = 0.338, P<0.01), HF (r = 0.332, P<0.05) and AF (r = 0.464, P<0.05) groups. Total hip BMD was correlated with SMI in the women HF group (r = 0.012, P<0.05), and in the men VF (r = 0.254, P<0.01) and HF (r = 0.197, P<0.05) groups; but these correlations were weak.

**Table 3:** Pearson’s correlation analysis of SMI and BMD in the different fracture groups and according to gender.

SMI (kg/m^2^)								
	Wrist Fracture		Vertebral fracture		Hip fracture		Ankle fracture	
	Female	Male	Female	Male	Female	Male	Female	Male
LSBMD (g/cm^2^)	0.035	0.111	0.076	0.439 b	0.187	0.636	0.094	0.344
FNBMD (g/cm^2^)	0.270 ^b^	0.546	0.159 ^a^	0.338 ^b^	0.337 ^b^	0.332 ^a^	0.022	0.464 ^a^
Total hip BMD (g/cm^2^)	0.359	0.544	0.222	0.254 ^b^	0.012^a^	0.197 ^a^	0.175	0.502

SMI = skeletal muscle index; LSBMD = lumbar spine bone mineral density; FNBMD = femoral neck bone mineral density; BMD = bone mineral density.

^a^: P<0.05

^b^: P<0.01

Logistic regression analyses were performed to determine the association of SMI and the occurrence of different fractures independently from the effect of BMD (Table 4). Lower SMI was associated with an increased risk of HF both in men and women (P = 0.007 and P = 0.008, respectively) and AF in men (P = 0.028). Higher Total Fat% was associated with an decreased risk of HF and VF in men (P = 0.001 and P = 0.012, respectively) an increased risk of AF in women (P = 0.001). In addition, younger age was associated with an increased risk of WF in women (P = 0.001), and a reduced risk of HF in both man and woman(P = 0.001 and P = 0.003, respectively).

**Table 4:** Logistic regression analysis of SMI and fracture risk stratified to sex.

	Wrist Fracture			Vertebral fracture			Hip fracture			Ankle fracture		
	OR	95%CI	P	OR	95%CI	P	OR	95%CI	P	OR	95%CI	P
**Women**												
**Age(years)**	0.917	0.872–0.964	0.001	1.001	0.958–1.066	0.498	1.130	1.044–1.224	0.003	0.920	0.884–1.959	0.788
**SMI(kg/m** ^**2**^ **)**	0.793	0.562–1,923	0.478	0.878	0.587–1.312	0.525	0.479	0.278–0.823	0.008	0.892	0.804–1.151	0.466
**Fat%**	1.003	0.934–1.139	0.583	0.950	0.958–1.041	0.809	0.976	0.921–1.034	0.409	1.096	1.044–1.151	0.001
**Men**												
**Age(years)**	1.007	0.743–1.126	0.378	1.023	0.972–1.213	0.801	1.079	1.020–1.207	0.001	0.977	0.923–1.035	0.430
**SMI(kg/m** ^**2**^ **)**	1.004	0.947–1.215	0.198	0.862	0.621–1.196	0.373	0.240	0.138–0.418	0.007	0.894	0.764–0.998	0.028
**Fat%**	0.925	0.794–1.013	0.185	0.934	0.885–0.985	0.012	0.867	0.794–0.947	0.001	0.947	0.894–1.005	0.069

Abbreviations: SMI = skeletal muscle index; Fat% = (TFM/weight)*100;OR = odds ratio; CI = confidence interval

## Discussion

The aim of the present study was to investigate the prevalence of sarcopenia in an elderly Chinese population and to assess the associations with a history of fragility fractures at various fracture locations. Results showed that sarcopenia was significantly more prevalent in men with WF, HF and AF than in women. SMI was correlated with BMD in VF, HF and AF. After adjustment for BMD, logistic regression analyses revealed that lower SMI was associated with an increased risk of HF in men and women, and AF in men.

Based on previous longitudinal studies, muscle mass decreases by around 40% between the age of 20 and 60 and leg lean tissue mass (assessed by DXA) decreases by about 1% per year.[23] However, there are differences in gender. Indeed, the absolute decrease is more pronounced in men than in women, though relative loss is comparable as men initially have more muscle mass.[24]

In this study, the prevalence of sarcopenia with HF was 41.9% in women and 84.1% in men. In the Sarcopenia and Hip Fracture study, 71% of participants were sarcopenic.[25] A recent Japanese study showed that 81.1% of male patients and 44.7% of female patients with HF were sarcopenic.[16] Another cross-sectional study of 591 inpatients after HF in Italy also showed this trend, but their results suggested a higher prevalence in both men and women (97% in men, 64% in women). These previous results support the high prevalence of sarcopenia in elderly patients with HF observed in the present study. Nevertheless, the differences in frequencies between the studies may be related to racial differences.[26]

Iolascon et al.[27] studied the prevalence of sarcopenia in women with VF. They observed that of the 52.23% women with VF, 22.85% were sarcopenic, and that of the 47.76% women with multiple VF, 43.75% were sarcopenic. In the present study, the prevalence of sarcopenia in women with VF was 33.9% in total, without considering single or multiple fractures. Recently a Japanese study [28] found women with acute osteoporotic vertebral fracture the OVF group showed lower appendicular SMI (5.62 vs. 5.97 kg/m^2^, P< 0.001), which was similar to the result of our study (5.89 vs. 6.27 kg/m^2^, P< 0.001)

There are several interrelationships between bone and muscle, and when the aging process affects one of these two tissues, the functionality of the other is compromised [7, 8, 29] Muscle and bone mass are both lost during aging starting in the late 20s and accelerating in the 50s.[30] Correlations between BMD and SMI were found in the present study in different fracture groups in both men and women, especially between FNBMD and SMI. A European study also showed that SMI was positively associated with BMD in men without adjustment.[6] SMI was significantly correlated with femoral BMD in another study of patients with HF.[31] Higher lean body mass was found to correlate to increased BMD and reduced fracture risk in menopausal women.[32]

Furthermore, in the present study, SMI was lower in the VF and HF female groups and in HF and AF male groups, compared with the NF group. Indeed, SMI was associated with an increased risk of HF in men and women, and AF in men. But the reason of inconformity in different fractures is still unknown. It may be related to many confounding and personal factors. Recently, a study has shown that decreased subcutaneous fat and SMI were associated with fracture risk in men (hazard ratio (HR) = 1.44, 95%CI: 1.02–2.02; and HR = 0.58, 95%CI: 0.36–0.91, respectively).[33] Since bone and muscle cells are derived from mesenchymal stem cells, they have similar genetic factors. Indeed, risk factors affecting osteoporosis and sarcopenia are heritable at approximately 60–70%.[29] In addition, bone and muscle share multiple endocrine factors including vitamin D, the growth hormone/IGF-1 axis and sex hormones [34]. Furthermore, mechanical stress exerted on bones is sensed by osteocytes that become activated and subsequently stimulate osteoblasts to increase BMD and bone strength at the site that is under most pressure.[35] Both muscle and fat tissue contribute to this mechanism, Previous studies had shown fat mass may have a protective role on osteoporosis even after adjusting for LM. [36, 37] In our study, higher total Fat% was associated with a decreased risk of HF and VF in men. However, the prevalence of obesity was significantly higher in AF group compared with NF groups in women. Likewise, a large examination of over 60,000 women also reported that high body weight is not protective against fracture incidence, but associated with ankle and upper leg fractures. [38] Furthermore, an increase in total and/or abdominal adipose tissue causes an increase in pro-inflammatory cytokines which led to a loss of skeletal muscle mass and function. [39, 40] Osteosaropeinc obesity (OSO) is recently identified and characterized by the simultaneous presence of osteopenia/osteoporosis, sarcopenia, and increased adiposity. [30, 34] Recently study has shown OSO related with the lowest handgrip scores, slowest normal and brisk walking speed, and shortest time for each leg stance, which could increase the risk of fall. [22] The prevalence of OSO in VF, HF and AF group were high than NF group in men and AF group in women in present study. Unfortunately we did not measure the function of the muscle and the risk of fall. In our opinion, appropriate fat mass may have positive effects on bone and reduce the risk of hip fracture. However, large fat mass lead to obesity, which may increase the risk of fall and inflammation, may have negative effects. This could explain the higher Fat% would increase risk of AF.

Although low correlation coefficients were obtained between BMD values (LSBMD, FNBMD, total Hip BMD) and the SMI for the assessed fracture sites, our results corroborate previous reports demonstrating the interrelationship between sarcopenia and BMD.[27, 28, 30, 41]

This study has several limitations. It is practically impossible to determine if sarcopenia actually caused the reported fractures, since the fractures could also result in decreased physical activity, and consequently, the loss of lean tissue. Since this work began in 2006, we did not collect data on muscle strength and physical performance as suggested by the ESPN in 2010.[3] As muscle strength declines much more rapidly than muscle mass, this data would have provided much important information for the conclusions of this study. Some patient information was missing such as the time of falls and fear of falling, comorbidities, complications and nutritional status, all of which may have a bearing on understanding the risk of fragility fracture. The time between when the fracture occurred to the DXA scan were unknown, and this information may affect the result of the study as the body composition measurements are unlikely to be static. We used strict inclusion criteria for selecting the patients in order to simplify our analysis, but as an elderly population is likely to have a high rate of poor health status, these may need to be less stringent to represent more accurately the aging population. In addition, we excluded patients who had been confined to a wheelchair or bed for a long time because these patients are more likely to suffer from disuse muscle atrophy. However, this may have affected the generalizability of the study. Further study would be worthwhile including the full history of the patients and full strength and physical performance evaluation with less stringent inclusion criteria.

In conclusion, this study showed the prevalence of sarcopenia and its association with different fragility fracture locations in elderly Chinese men and women for the first time. Sarcopenia was significantly more prevalent in men with WF, HF and AF than in women. SMI was correlated with BMD in VF, HF and AF. After adjustment for BMD, logistic regression analyses revealed that lower SMI was associated with an increased risk of HF in men and women and AF in men. Sarcopenia may be an independent risk factor for HF and AF in men, and for HF in women.

## References

[1] Cederholm T, Cruz-Jentoft AJ, Maggi S. Sarcopenia and fragility fractures. Eur J Phys Rehabil Med. 2013;49: 111–117. 23575205

[2] Dhanwal DK, Dennison EM, Harvey NC, Cooper C. Epidemiology of hip fracture: Worldwide geographic variation. Indian J Orthop. 2011;45: 15–22. 10.4103/0019-5413.73656 21221218 PMC3004072

[3] Cruz-Jentoft AJ, Baeyens JP, Bauer JM, Boirie Y, Cederholm T, Landi F, et al Sarcopenia: European consensus on definition and diagnosis: Report of the European Working Group on Sarcopenia in Older People. Age Ageing. 2010;39: 412–423. 10.1093/ageing/afq034 20392703 PMC2886201

[4] Baumgartner RN, Koehler KM, Gallagher D, Romero L, Heymsfield SB, Ross RR, et al Epidemiology of sarcopenia among the elderly in New Mexico. Am J Epidemiol. 1998;147: 755–763. 9554417 10.1093/oxfordjournals.aje.a009520

[5] Fried LP, Tangen CM, Walston J, Newman AB, Hirsch C, Gottdiener J, et al Frailty in older adults: evidence for a phenotype. J Gerontol A Biol Sci Med Sci. 2001;56: M146–156. 11253156 10.1093/gerona/56.3.m146

[6] Verschueren S, Gielen E, O'Neill TW, Pye SR, Adams JE, Ward KA, et al Sarcopenia and its relationship with bone mineral density in middle-aged and elderly European men. Osteoporos Int. 2013;24: 87–98. 10.1007/s00198-012-2057-z 22776861

[7] Edwards MH, Dennison EM, Aihie Sayer A, Fielding R, Cooper C. Osteoporosis and sarcopenia in older age. Bone. 2015. 10.1016/j.bone.2015.04.016 PMC4601530 25886902

[8] Tarantino U, Piccirilli E, Fantini M, Baldi J, Gasbarra E, Bei R. Sarcopenia and fragility fractures: molecular and clinical evidence of the bone-muscle interaction. J Bone Joint Surg Am. 2015;97: 429–437. 10.2106/JBJS.N.00648 25740034

[9] Di Monaco M, Castiglioni C, Vallero F, Di Monaco R, Tappero R. Sarcopenia is more prevalent in men than in women after hip fracture: a cross-sectional study of 591 inpatients. Arch Gerontol Geriatr. 2012;55: e48–52. 10.1016/j.archger.2012.05.002 22647380

[10] Chen LK, Liu LK, Woo J, Assantachai P, Auyeung TW, Bahyah KS, et al Sarcopenia in Asia: consensus report of the Asian Working Group for Sarcopenia. J Am Med Dir Assoc. 2014;15: 95–101. 10.1016/j.jamda.2013.11.025 24461239

[11] Cooper C, Dere W, Evans W, Kanis JA, Rizzoli R, Sayer AA, et al Frailty and sarcopenia: definitions and outcome parameters. Osteoporos Int. 2012;23: 1839–1848. 10.1007/s00198-012-1913-1 22290243

[12] Wang ZM, Visser M, Ma R, Baumgartner RN, Kotler D, Gallagher D, et al Skeletal muscle mass: evaluation of neutron activation and dual-energy X-ray absorptiometry methods. J Appl Physiol (1985). 1996;80: 824–831. 8964743 10.1152/jappl.1996.80.3.824

[13] Cummings SR, Melton LJ. Epidemiology and outcomes of osteoporotic fractures. Lancet. 2002;359: 1761–1767. 12049882 10.1016/S0140-6736(02)08657-9

[14] Akesson K, Marsh D, Mitchell PJ, McLellan AR, Stenmark J, Pierroz DD, et al Capture the Fracture: a Best Practice Framework and global campaign to break the fragility fracture cycle. Osteoporos Int. 2013;24: 2135–2152. 10.1007/s00198-013-2348-z 23589162 PMC3706734

[15] Demontiero O, Gunawardene P, Duque G. Postoperative prevention of falls in older adults with fragility fractures. Clin Geriatr Med. 2014;30: 333–347. 10.1016/j.cger.2014.01.018 24721372

[16] Hida T, Ishiguro N, Shimokata H, Sakai Y, Matsui Y, Takemura M, et al High prevalence of sarcopenia and reduced leg muscle mass in Japanese patients immediately after a hip fracture. Geriatr Gerontol Int. 2013;13: 413–420. 10.1111/j.1447-0594.2012.00918.x 22816427

[17] Cheng Q, Zhu X, Zhang X, Li H, Du Y, Hong W, et al A cross-sectional study of loss of muscle mass corresponding to sarcopenia in healthy Chinese men and women: reference values, prevalence, and association with bone mass. J Bone Miner Metab. 2014;32: 78–88. 10.1007/s00774-013-0468-3 23620096

[18] World Health Organization (1998) Guidelines for preclinical evaluation and clinical trials in osteoporosis. Geneva: World Health Organization.

[19] Heymsfield SB, Smith R, Aulet M, Bensen B, Lichtman S, Wang J, et al Appendicular skeletal muscle mass: measurement by dual-photon absorptiometry. Am J Clin Nutr. 1990;52: 214–218. 2375286 10.1093/ajcn/52.2.214

[20] Kanis JA, Melton LJ III, Christiansen C, Johnston CC, Khaltaev N. The diagnosis of osteoporosis. J Bone Miner Res. 1994;9: 1137–1141. 7976495 10.1002/jbmr.5650090802

[21] American Society of Bariatric Physicians (2005) Obesity algorithm.

[22] Ilich JZ, Inglis JE, Kelly OJ, McGee DL. Osteosarcopenic obesity is associated with reduced handgrip strength, walking abilities, and balance in postmenopausal women. Osteoporos Int. 2015. 10.1007/s00198-015-3186-y 26025288

[23] Lang T, Streeper T, Cawthon P, Baldwin K, Taaffe DR, Harris TB. Sarcopenia: etiology, clinical consequences, intervention, and assessment. Osteoporos Int. 2010;21: 543–559. 10.1007/s00198-009-1059-y 19779761 PMC2832869

[24] Gallagher D, Visser M, De Meersman RE, Sepulveda D, Baumgartner RN, Pierson RN, et al Appendicular skeletal muscle mass: effects of age, gender, and ethnicity. J Appl Physiol (1985). 1997;83: 229–239. 9216968 10.1152/jappl.1997.83.1.229

[25] Fiatarone Singh MA, Singh NA, Hansen RD, Finnegan TP, Allen BJ, Diamond TH, et al Methodology and baseline characteristics for the Sarcopenia and Hip Fracture study: a 5-year prospective study. J Gerontol A Biol Sci Med Sci. 2009;64: 568–574. 10.1093/gerona/glp002 19228788

[26] Di Monaco M, Vallero F, Di Monaco R, Tappero R. Prevalence of sarcopenia and its association with osteoporosis in 313 older women following a hip fracture. Arch Gerontol Geriatr. 2011;52: 71–74. 10.1016/j.archger.2010.02.002 20207030

[27] Iolascon G, Giamattei MT, Moretti A, Di Pietro G, Gimigliano F, Gimigliano R. Sarcopenia in women with vertebral fragility fractures. Aging Clin Exp Res. 2013;25 Suppl 1: S129–131. 10.1007/s40520-013-0102-1 24046029

[28] Hida T, Shimokata H, Sakai Y, Ito S, Matsui Y, Takemura M, et al Sarcopenia and sarcopenic leg as potential risk factors for acute osteoporotic vertebral fracture among older women. Eur Spine J. 2015. 10.1007/s00586-015-3805-5 25690348

[29] Karasik D, Kiel DP. Genetics of the musculoskeletal system: a pleiotropic approach. J Bone Miner Res. 2008;23: 788–802. 10.1359/jbmr.080218 18269309 PMC3280426

[30] Ormsbee MJ, Prado CM, Ilich JZ, Purcell S, Siervo M, Folsom A, et al Osteosarcopenic obesity: the role of bone, muscle, and fat on health. J Cachexia Sarcopenia Muscle. 2014;5: 183–192. 10.1007/s13539-014-0146-x 24740742 PMC4159494

[31] Di Monaco M, Vallero F, Di Monaco R, Tappero R, Cavanna A. Skeletal muscle mass, fat mass, and hip bone mineral density in elderly women with hip fracture. J Bone Miner Metab. 2007;25: 237–242. 17593494 10.1007/s00774-007-0752-1

[32] Kaji H. Linkage between muscle and bone: common catabolic signals resulting in osteoporosis and sarcopenia. Curr Opin Clin Nutr Metab Care. 2013;16: 272–277. 10.1097/MCO.0b013e32835fe6a5 23481148

[33] Malkov S, Cawthon PM, Peters KW, Cauley JA, Murphy RA, Visser M, et al Hip Fractures Risk in Older Men and Women Associated with DXA-Derived Measures of Thigh Subcutaneous Fat Thickness, Cross-Sectional Muscle Area, and Muscle Density. J Bone Miner Res. 2015. 10.1002/jbmr.2469 PMC5111546 25644748

[34] Ilich JZ, Kelly OJ, Inglis JE, Panton LB, Duque G, Ormsbee MJ. Interrelationship among muscle, fat, and bone: connecting the dots on cellular, hormonal, and whole body levels. Ageing Res Rev. 2014;15: 51–60. 10.1016/j.arr.2014.02.007 24632496

[35] Rochefort GY, Pallu S, Benhamou CL. Osteocyte: the unrecognized side of bone tissue. Osteoporos Int. 2010;21: 1457–1469. 10.1007/s00198-010-1194-5 20204595

[36] Oros S, Ianas O, Vladoiu S, Giurcaneanu M, Ionescu L, Neacsu E, et al Does Obesity Protect Postmenopausal Women Against Osteoporosis? Acta Endocrinologica (1841–0987). 2012;8: 67–76.

[37] Kim W, Chung SG, Kim K, Seo HG, Oh BM, Yi Y, et al The relationship between body fat and bone mineral density in Korean men and women. J Bone Miner Metab. 2014;32: 709–717. 10.1007/s00774-013-0545-7 24374493

[38] Compston JE, Watts NB, Chapurlat R, Cooper C, Boonen S, Greenspan S, et al Obesity is not protective against fracture in postmenopausal women: GLOW. Am J Med. 2011;124: 1043–1050. 10.1016/j.amjmed.2011.06.013 22017783 PMC4897773

[39] Li Z, Heber D. Sarcopenic obesity in the elderly and strategies for weight management. Nutr Rev. 2012;70: 57–64. 10.1111/j.1753-4887.2011.00453.x 22221216

[40] Malafarina V, Uriz-Otano F, Iniesta R, Gil-Guerrero L. Sarcopenia in the elderly: diagnosis, physiopathology and treatment. Maturitas. 2012;71: 109–114. 10.1016/j.maturitas.2011.11.012 22153348

[41] Hita-Contreras F, Martinez-Amat A, Cruz-Diaz D, Perez-Lopez FR. Osteosarcopenic obesity and fall prevention strategies. Maturitas. 2015;80: 126–132. 10.1016/j.maturitas.2014.11.009 25533145

